# Medical tourism among Indonesians: a scoping review

**DOI:** 10.1186/s12913-023-10528-1

**Published:** 2024-01-10

**Authors:** Gregorius Abanit Asa, Nelsensius Klau Fauk, Caitlan McLean, Paul Russell Ward

**Affiliations:** 1 Research Centre for Public Health, Equity and Human Flourishing (PHEHF), Torrens University Australia, Adelaide, South Australia Australia; 2 Institute of Resource Governance and Social Change, Kupang, Indonesia

**Keywords:** Scoping review, International medical travel, Medical tourism, Medical treatment, Indonesia

## Abstract

**Background:**

International medical travel or medical tourism is not a new phenomenon in many countries, including among Indonesians. Indonesia is reported as a major source of patients from the lower, middle, to upper classes for its neighbouring countries. This scoping review aims to synthesise evidence on supporting factors for Indonesians taking medical tourism and what needs to be improved in Indonesia’s health system.

**Methods:**

We conducted a scoping review guided by a framework provided by Arksey and O’Malley. We systematically searched existing literature from 5 databases, including MEDLINE, PubMed, Scopus, ProQuest, and Wiley. Data were extracted based on study details, study design, characteristics of participants and results. Analysis followed the three-stage procedure outlined by Thomas and Harden: (1) coding the text line by line, interpreting the data and identifying concepts or themes; (2) developing descriptive themes by grouping similar concepts in theme and subtheme and (3) generating analytical themes by reviewing preliminary themes and discussing the addition or revision of themes.

**Results:**

A total of 25 articles were included in this review. The review highlights a broad range of facilitators for medical tourism among Indonesians: (i) availability of health services, medical specialities, and person-centred care, (ii) region adjacency, transport, and health agency, (iii) affordability of medical treatment, (iv) religious and socio-cultural factors, and (v) reasons patients reported distrust in Indonesian doctors.

**Conclusion:**

The findings indicate improvements in the Indonesian health system are necessary if the increasing rates of international medical tourism by Indonesian people are to change. Addressing the factors identified in this scoping review through avenues including policy may increase people’s satisfaction and trust towards health care and treatment in Indonesia, thereby reducing the number of Indonesian people taking medical tourism.

**Supplementary Information:**

The online version contains supplementary material available at 10.1186/s12913-023-10528-1.

## Introduction

International medical travel or medical tourism is not a new phenomenon. It refers to the practice of patients travelling overseas for better medical treatment and relaxation [1–3] and can be traced back to the ancient times when Greek pilgrims travelled from the Mediterranean Sea to Epidaurus, a small territory known as the healing god [4]. People around the world have travelled to India for Yoga and Ayurvedic healing since the 1500s [4]. In the 18th and 19th centuries, Europeans travelled to spa towns in the south of France to treat their diseases as well as to enjoy the sun and escape from cold climatic condition [5]. Since the 19th century, more people have taken international medical travel to treat their diseases [6]. Medical tourism is correspondent with the growth of global health services, marked by increasing international trade in health products [7].

Available reports have suggested a significant increase in the volume of international medical travel has occurred since the late 1990s [7], ranging from thousands to millions every year [8, 9]. Asia is among the biggest players driving international medical travel in affordable and high-quality care [10]. Some Asian countries, such as South Korea, Singapore, Malaysia, Thailand, and India, are considered the major international medical travel destinations [11–14]. Thailand has been known as a medical tourism destination since the 1970s [15]. Malaysia and Singapore have reformed the healthcare system since the 1980s, resulting in improved health quality services attracting people from neighbouring countries [16]. This is also supported by the advancement of infrastructure and technologies, allowing people to access health services easily and to be well-informed about global health. In the context of Asia, the development of international medical tourism was partly pushed by the Asian financial crisis in the 1990s, particularly caused difficulties and reluctance among many middle class Asia to access private healthcare, resulting in private hospitals generating new sources of revenue by targeting international patients [11, 17].

Indonesians have been taking international medical travel to neighbouring countries with better healthcare services for many years. The range of Indonesians health condition treated in neighbouring countries include cardiology treatment (bypass surgery and angioplasty), orthopaedic procedures (knee and hip surgery), cancer treatment (chemotherapy and radiotherapy), cosmetic and plastic surgery procedures (breast augmentation and facelifts), fertility treatment (in-vitro fertilisation and intrauterine insemination), dental treatments, ophthalmology procedures, neurosurgery procedures (brain tumours and spinal surgeries), and urology treatments (kidney stone removal and prostate surgery) [18–22]. Studies have reported that Indonesia is a major source of patients from the lower, middle, to upper classes for its neighbour countries [23–25] and has been the primary revenue contributor for Malaysian (> 75%) and Singapore (60%) medical tourism [26, 27]. It is reported that nearly two million Indonesians travelled overseas for medical treatment in 2022 [28]. Of these, about 1 million travelled to Malaysia, 750,000 to Singapore, and the rest to Japan, the US, Germany and other countries, resulting in Indonesia losing 11.5 billion US Dollars (IDR 170 trillion) annually [28, 29]. It is also reported that about 60% of Indonesians who took international medical travel were from Jakarta, 15% were from East Java, and the rest from other cities such as Medan, Batam, and Kalimantan [29]. Such loss has attracted the Indonesian government’s attention to curb international medical travel, which has started since 2010 by improving health facilities in West Kalimantan to stem people in West Kalimantan from going to Malaysia [30]. For example, the government invested USD 660,000 to provide magnetic resonance imaging (MRI) and computed tomography (CT) scanners in the public Regional General Hospital Soedarso in Pontianak [30]. In 2012, an agreement between The Ministry of Tourism and Creative Economy and the Ministry of Health was made as part of the effort to strengthen the partnership with Bali International Medical Centre (BIMC) Hospital and the Courtyard by Marriott Bali Hotel and construct a new hospital equipped with world-class facilities in Sanur, Bali in 2016 [23].

Internationally, studies have suggested push and pull factors to explain why patients travel internationally for their medical care [31–34]. Factors included the cost of care in a person’s home country [35–38] as well as patients’ concerns about quality of services, care, facilities and a lack of qualified doctors [33, 34]. Some findings have also suggested that patients’ decisions for medical tourism are influenced by the availability and ease of travel and transportation to the designated countries and better procedures to access medical treatment [8]. Perceptions of faster and more convenient services in other countries, and distrust in doctors in home countries are also supporting factors for patients’ medical tourism [39].

Although there have been some reviews on international medical travel in some settings [8, 39], there have been no reviews in the context of Indonesia. The authors considered it important to conduct a scoping review to synthesise evidence on reasons or supporting factors for Indonesians travelling overseas for medical treatment. The review was conducted to address a specific question: what factors facilitate Indonesian patients seeking medical treatment overseas? To determine whether there have been any previous reviews exploring topic of medical tourisms among Indonesians, we conducted a preliminary search in PubMed and CINHAL and found no ongoing or published reviews. This scoping review seeks to identify how Indonesia’s existing health care system could be improved to reduce international medical tourism.

## Methods

### Design

To identify available evidence on the topic, we apply several steps suggested in a framework by Arksey and O’Malley and additional recommendations from Levac and colleagues [40, 41]: (1) identifying the research question, (2) identifying relevant studies, (3) study selection, (4) charting the data, and (5) accumulating, summarizing, and reporting the results. To guide the search strategy, this review aims to answer the following question: “what are the facilitators for Indonesians taking international medical travel?”.

### Search strategy for identifying relevant studies

We developed inclusion and exclusion criteria to guide the search and selection of studies for this review (outlined in Table 1). The search included studies from 2000 to July 2023. This timeframe was justified as it aligns with an increase in the rate of international medical travel [25, 34]. Data search was conducted from 1 to 10 August 2023 in the following databases: MEDLINE, PubMed, Scopus, ProQuest, and Wiley. These databases were chosen as they are among large citation databases providing access to literatures on health and tourism. The following key concepts were used across the databases: international medical travel/medical tourism, patients/travellers, facilitators, and Indonesian. We developed synonyms of the key concepts for the search. A full description of the search terms used for each database is in the supplementary file 1. The search terms were formulated using the combination of key terms or the synonym of each concept using Boolean terms (OR and AND). We also searched grey literature using the key concepts in google scholar and Google to increase comprehensiveness searching of available evidence. Data from across the databases was exported to Endnote and all duplicates were removed. The authors then screened the articles based on the title and abstract. The researchers (GAA and NKF) completed independent screening and blindly labelled each study according to inclusion and exclusion criteria. Disagreements were resolved by all authors. We also did manual searches of the reference lists of all studies included after screening. An example of a complete search string in Scopus is provided below:

“International medical travel” OR “medical tourism” OR “health tourism” OR “health travel” AND facilitators OR “supporting factors” OR reasons OR “push factors” OR “pull factors” AND patients OR travellers AND indonesia* OR indonesian.

**Table 1:** inclusion and exclusion criteria

Criterion	Inclusion criteria	Exclusion criteria
Population	Indonesian patients: men, women, adult, and elderly with any disease.	
Phenomenon of interest	Indonesians taking international medical travel	Diasporic Indonesians seeking medical treatment in Indonesia, Indonesian patients from living in other countries, and Indonesian health workers living and working in other countries
Context	Travel to other developing or developed countries	Travel within country
Study design	Quantitative method, qualitative method, mixed method, review, systematic review, and published in peer reviewed journals. Grey literature including conference papers, thesis, book chapters, and report	
Language	English and Indonesia	Articles published other than in English and Indonesia
Purpose of study	Studies aiming at exploring facilitators for Indonesians taking international medical treatment	
Text	Full text available	Abstract only
Year publication	2000–2023	Studies outside these dates

### Study selection and screening

**Fig. 1:**
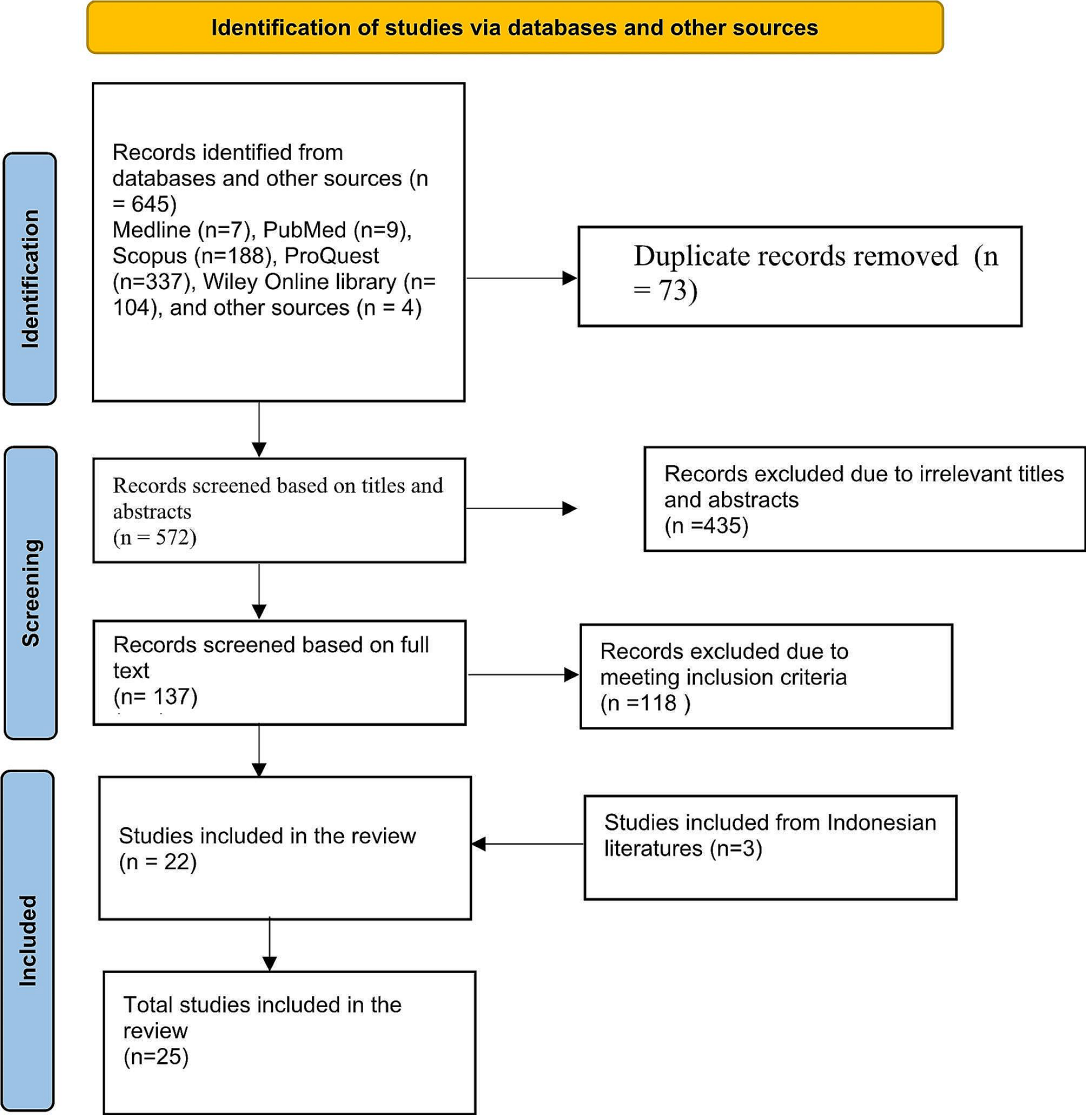
PRISMA Flow diagram of systematic literature search: records identified, screened, eligible and included in the review

Using developed search terms, 649 articles were identified. Of these, 75 were removed due to duplication in endnote software, leaving 572 articles. The remaining articles were screened according to titles and abstracts, resulting in removing 437 articles. We then screened the full texts of the remaining 137 articles. Of these, 118 articles were excluded due to not meeting inclusion criteria. Throughout the screening process, any disagreements were examined through discussion among authors, resulting in 22 articles being included. Three articles were found from the references of the previous literatures, and 3 literatures in Indonesian language were found through google scholar. Finally, 25 literatures were included in the scoping review (Fig. 1 full article screening process).

### Data extraction and synthesis (charting the data)

An extraction sheet was developed and used to extract the following information from each study: (1) study details: the last name of the first author, year of publication; (2) study design: type of study, study aim and analysis methods; (3) analysis, and (4) results (Supplementary File 2). Data extraction was conducted by GAA and NKF and results were read and discussed by all team members.

### Data analysis

Analysis followed the three-stage procedure outlined by Thomas and Harden: [42, 43] (1) coding the text line by line, interpreting the data and identifying concepts or themes; (2) developing descriptive themes by grouping similar concepts in theme and subtheme and (3) generating analytical themes by reviewing preliminary themes and discussing the addition or revision of themes. Finally, the major themes relating to Indonesian people undertaking international medical travel were identified and discussed.

## Result

### Characteristics of included studies

An overview of the characteristics of the included studies can be found in the supplementary file 2. Of the 25 articles discussed in the review, 10 studies used qualitative methods [35–37, 44–50], 12 studies used quantitative methods [20–22, 38, 51–58]. Additionally, a mixed-method study [59], a conference review [60], and a review [23] were included. The studies included began from 2011 until 2022. 21 studies discussed Indonesian patients accessing medical treatment in Malaysia [20–23, 35–37, 39, 44–49, 51, 52, 55, 57–60], 4 studies examined Indonesian patients accessing medical treatment in Singapore [23, 36, 56, 60], and 3 of the studies examined Indonesian patients accessing medical treatment in Thailand [23, 35, 36]. 22 articles were published in English [20–22, 35–38, 44–54, 58], and 3 articles were published in Indonesian language [55–57]. Most participants were from middle to upper class background [20, 21, 35, 37, 38, 44, 47, 54, 58, 61]. Participants from low economic status were reported in two studies [45, 54]. The occupation of the participants included housewife, student, driver, office clerk, civil servant, professional, business owner, and retired [21, 44, 45, 49, 51]. The participants were from Jakarta, Surabaya, Yogyakarta, Bali, West Nusa Tenggara, North Sumatra, West Java, Nort Sulawesi, Riau, West Sumatra, Aceh, Bangka, and Kalimantan [35–37, 44–48, 54, 56, 58].

The review of the included studies is discussed in the following themes: (1) availability of health services, medical specialities, and person-centred care: (2) region adjacency, transport, and health agency: (3) affordability of medical treatment: (4) religious and socio-cultural factors: and (5) reasons patients reported distrust in Indonesian doctors.

### Theme 1: availability of health services, medical specialities, and person-centred care

#### Health facilities and medical specialists

Several studies reported that the availability of needed medical treatment, advanced medical technology, and medical specialists for specific health issues in other countries, which are not available in Indonesia have been amongst other main reasons for Indonesians travelling overseas for medical treatment [20, 23, 35, 36, 38, 45, 47, 53, 60]. For example, a study with elderly mothers in Medan, North Sumatra reported a lack of medical equipment for heart and stroke in the setting, leading some Medanese taking international medical travel to Penang, Malaysia for medical treatment [35]. Similarly, another study with married infertile Indonesian couples suggested that the unavailability of assisted reproduction technologies (ART) in healthcare facilities Indonesia is a supporting factor medical tourism among the Indonesian couples [36]. In addition, sperm and egg donation as well as surrogacy are strictly banned in Indonesia, giving Indonesian couples no choice but to travel overseas to access the required health service. Lack of medical specialists is another challenge in healthcare facilities in periphery regions of Indonesia. Healthcare facilities in periphery of Indonesia is reported to experience shortage of qualified doctors, and difficulties to retain qualified doctors for long term, leading patients to seeking qualified doctors overseas or in neighbouring countries, such as Malaysia and Singapore [45]. Such situations are emphasised in Mahendradhata’s study on challenge and risks for healthcare tourism, suggesting that medical specialists, health facilities, and health technicians outside Jakarta are inadequately and unequally distributed, resulting in Indonesians seeking alternative medical treatment overseas [23].

The knowledge and experiences of well-organised and fast healthcare services in other countries, such as Malaysia and Singapore are also reported as factors attracting Indonesian patients to have medical treatment overseas [23, 37, 44, 45, 47, 51, 62]. For example, a study reported that it took one day with details clearly explained in a hospital in Penang, Malaysia to be informed about the results of blood test, and four days in Indonesia to receive the results of the same test [37]. Also, long queues for consultation with medical specialists and disorganised medical records were detrimental for patients’ health, resulting in patients making decision to have medical treatment overseas [62]. The experiences of complex medical processes and unnecessary requirements had also resulted in disappointment with the hospitals and their services and patients seeking medical treatment overseas [45]. Meanwhile, the experience of simple ease of making medical appointments with overseas hospitals or doctors from Indonesia through smartphones is also another supporting factor for medical tourism in among Indonesians [37].

#### Person-centred care

Several studies reported lack of person-centred care as a supporting factor for Indonesian patients seeking medical treatment overseas [45, 47, 59, 62]. A study with Indonesian patients in Malaysia and Singapore suggested that patients experienced the feelings of care, respect and positive self-esteem through positive and supporting attitudes and behaviours of medical staffs towards them and their family members during medical treatment [45, 59]. Such positive attitudes and behaviours of medical staff are reflected in being cheerful, and smiling while serving, and good communication with patients and their families, which also lead to positive patient-doctor relations [62]. Thus, studies with Indonesian patients accessing medical treatment in these countries suggested patients felt their dignity being maintained and respected and no frustration due to feeling unimportant or ignored [47].

### Theme 2: region adjacency, transport, and health agency

#### Geographical and transport factors

A few studies described several provinces in Indonesia are geographically closer to Malaysia and Singapore than to Jakarta as a supporting factor for Indonesians taking international medical travel to these countries [35, 37, 38, 44, 54]. Indonesian patients accessing medical treatment in Penang (Malaysia), were not only from North Sumatra and Aceh but also from Jakarta, East Java, West Java, and other regions in Sumatra [54]. Patients from Medan and Aceh in Indonesia stated that geographical proximity to Penang-Malaysia and reliability of transport through regular flights and ferries were supporting factors for them taking international medical travel [35]. These factors were reported to create much more comfortable feeling for Indonesian patients to access medical treatment in Malaysia than in Jakarta, Indonesia [44]. In addition, some studies reported that Indonesian patients from Kalimantan reached healthcare facilities in Kuching, Malaysia, by bus, taxi and uber (Grab) which are more economical than flying to Jakarta [37, 41, 44]. Taxi drivers were also reported to have knowledge of hospitals for international travellers as they often drove patients from overseas. Taxi drivers shared knowledge such as availability of specialised doctors, further supporting Indonesian patients seeking medical treatment in Malaysia [44].

#### Health agency

It was also reported that several Malaysian private hospitals have their official offices in Pontianak, Indonesia, that helped register customers, schedule consultations, and manage complaints [45]. This was also supported by contractual business between hospitals in Malaysia and certain local companies in Indonesia, acting as “medical representatives” that help facilitate patients to use medical care overseas. Such systems result in successfully recruited an average of 5000 patients per month [48]. A quantitative study found more than 90% of Indonesians travelling to Malaysia for medical treatment were influenced by marketing promotion programs including word of mouth, advertisements, sales promotion, and public relations [58]. The regular visitations of Malaysian doctors to Indonesia and holding health exhibition and public talks in temples, churches, and mosques in Indonesia, which introduce and disseminate medical treatment in Malaysia to the Indonesian, were also supporting factors for medical tourism among Indonesians [48].

### Theme 3: affordability of medical treatment

Most of the included studies found that medical treatments in Jakarta and in Kalimantan were more expensive than in Malaysia, another supporting factor for medical tourism for Indonesian people [20, 36–38, 44, 45, 51–53, 60, 49]. For example, chemotherapy in Penang, Malaysia was reportedly cheaper than in Jakarta and Medan, Indonesia [37]. Patients were reported to receive free health consultation following medical treatment in Malaysia [52]. Meanwhile, patients in Indonesia tended to be asked for frequent paid visits and consultations with doctors, leading to increased medical costs [44].

For some Indonesian lower middle class patients, accessing medical treatment overseas was acknowledged to have additional financial burdens for transports and accommodations, however such treatment was still considered worthwhile compared to having treatment in Indonesia [45, 46]. While for some Indonesian upper class patients, it is their preference to have medical treatment in Singapore and Thailand due to excellent health system and a very high quality of medical care, irrespective of the cost being more expensive in Singapore than Malaysia [36].

### Theme 4: religious and socio-cultural factors

Studies also reported that the preference to have medical treatment overseas was also influenced by religious reasons, cultural background, and their attitudes towards private and public hospitals in Indonesia [35, 36, 38, 60]. For example, a study suggested that Indonesian Muslim patients accessed In Vitro Fertilization (IVF) in Malaysia due to compatible religious backgrounds feeling safer to be treated by Muslim doctors who knew about halal and haram in Islam law [36]. Similarly, Chinese Indonesian patients felt comfortable seeking medical treatment in Singapore due to its ethnic Chinese majority [36]. In addition, having a sense of class difference to native Indonesians and the perceptions that Indonesian government hospitals were for native Indonesians, were also reported as supporting factors for Chinese Indonesian patients to choose private hospitals overseas [35]. Another supporting factor for medical tourism among Indonesia patients is the sense of self-fulfilment for prestige it provides [53].

Social support from others (i.e., families, friends, and neighbours) through the provision of information about medical treatment overseas was also an enabling factor for Indonesians’ decisions to take international medical travel [21, 22, 44, 45, 47–49]. For example, it was reported that around 60% of Indonesian patients took medical travel to Malaysia following recommendation from their families and friends who had either visited or lived there, while some acquired information through the internet (about 14%) and travel agents (around 12%) [21]. Other findings have also suggested that former Indonesian patients tended to share their experiences and recommended the services of medical specialists in Malaysia to their friends and family [45]. This is also supported by communication skills of Malaysian doctors in using Chinese dialects when communicating with older Chinese Indonesians [45].

Language similarities which create easy communication between Indonesian patients and other people or healthcare professionals in Malaysia were also contributing factors for the state of comfort for patients [37, 45, 59]. These were reported to make it easier for Indonesian patients to discuss and describe their health issues to healthcare professionals in Malaysia [37, 45, 59]. In addition, studies also reported that Indonesian patients who travel to Singapore and Thailand were from upper class background and were proficient with English and Chinese languages [37, 59].

### Theme 5: reasons patients reported distrust in Indonesian doctors

Most of the included studies reported a lack of trust in healthcare professionals as an influencing factor for Indonesian patients travelling overseas for medical treatment [35, 37, 44–47, 53, 49, 63]. A study with patients in Pontianak, Kalimantan reported patients’ comparison of ineffective medications prescribed by Indonesian doctors in Pontianak and in Jakarta with medication received from doctors in Kuching, Malaysia, proving to bring positive progress to their health condition [44]. The ineffective medication was highlighted as the reasons for their multiple visits and consultations with medical doctors in Indonesia, which decreased their trust in the Indonesian doctors and supported their decision for medical tourism [44]. Patients’ distrust in Indonesian doctors (i.e., in Pontianak, Kalimantan) was also evidenced by the lack of accuracy in health issue diagnosis, leading to patients seeking a second opinion from doctors overseas and receiving different diagnosis results and treatment and experiencing positive health recovery [37, 44]. For some patients, the lack of diagnosis accuracy and ineffective medication had led to long period of medical treatments in Indonesia without progress, which was a supporting factor for them seeking treatment overseas [44]. A couple of studies have suggested criticism and suspicion held by patients towards Indonesian doctors in Pontianak-Kalimantan. This distrust was linked prescription of a range of all antibiotics to patients without accurate diagnosis of the health issue and the tendency for doctors to overstate or exaggerate patients’ diseases [44, 45]. For example, a patient had been scheduled for appendix surgery in Indonesia but was diagnosed with simple constipation in Penang, Malaysia [35]. The lack of trust and confidence in the Indonesian doctors in some settings had led to Indonesian patients travelling to neighbouring countries for medical treatment. They felt that doctors overseas provided clear information regarding the disease and the percentage of likelihood for a cure while patient’s in the studies reported Indonesian doctors in Pontianak were reported to sometimes hide the truth from them [45, 54].

Distrust in doctors has been reported to lead to patients visiting Indonesian doctors only for ‘small things’ or health issues and considering taking international medical travel as a better option [35]. This is in line with another study reporting that some Indonesian patients did not dare to take risk for operation or surgery in Indonesia due to fear of malpractice or failed operation which may lead to negative outcomes including paralysis [46]. Negative perceptions towards doctors in Indonesia were also attributed to a claim that doctors easily made money via prescribing varieties of medicine for patients to consume which may potentially have high risks of overdoses [47]. Similarly, another study found that health professionals in Indonesia were reported as being arrogant, incompetent and untrustworthy, leading patients to express disdain to hierarchical medical culture that seemingly positions patients as passive consumers rather than active recipients [63].

## Discussion

To the best of our knowledge, this is the first scoping review to synthesise the available evidence on factors supporting international medical travel from Indonesia. It is noted that the number of Indonesian patients from low, middle, and upper class participating in international medical travel has increased in recent years [28, 53, 64]. Our findings suggest that international medical travel by Indonesian patients is linked to five domains:(i) the availability of health services, medical specialities, and person-centred care in the designated countries, (ii) region adjacency, transport, and health agency, (iii) affordability of medical treatment in other countries, (iv) religious and socio-cultural factors, and (v) reasons patients reported distrust in Indonesian doctors.

Overall, our findings have highlighted that patient’s perceive low quality of Indonesian health care and treatment, resulting in Indonesia becoming the major supplier of patients to neighbouring countries, such as Malaysia, Singapore, and Thailand [23–25]. This scoping review shows that patients seeking medical treatment outside of Indonesia do so due to a number of reported issues, including the unavailability of medical equipment, inadequate qualified doctors, and inadequate trained staff in healthcare facilities in remote and border areas of Indonesia. These factors have been identified as heavily influencing patients’ preferences to seek medical treatment overseas. This scoping review supports previous findings which have reported that Indonesia has a comparably low ratio of qualified doctors to patients, and most prefer to work in private hospitals in urban areas within Indonesia [65–67], resulting in understaffing and the maldistribution of skilled staff within periphery areas [68, 69]. It is suggested that with a population of about 270 million, Indonesia needs 270,000 doctors [70]. Currently, Indonesia has only 110,000 doctors with the ratio of doctors to patients being 0.6:1000, which is very low compared to other countries, such as Malaysia with the ratio of 2.2:1000 [70].

Across studies, patients reported feeling frustrated, neglected and that they were not being provided with person-centred care within Indonesian health settings. Further, evidence suggests patients felt undervalued in Indonesian health systems and that their care was not prioritised. This was identified as stemming from factors including sparseness of health facilities, a shortage of qualified doctors, and trained staffs [69, 71]. There is also evidence that patients sought more timely health care and treatment overseas as a consequence of long waiting time periods for medical treatment within Indonesia [72].

Reflecting upon geographical proximity, it is understandable that inequality of health facilities and medical staff distribution between Java and border areas or eastern part of Indonesia has contributed to patients’ decision to take international medical travel to the nearest neighbouring countries. Some regions in Malaysia such as Penang, Melaka, and Kuching are the most popular destinations for Indonesian patients. For example, Kuching can be accessed by air taking about 45 min and by land taking about 10–12 h, allowing lower-middle class patients from West Kalimantan to easily have access to medical services [35, 37, 38, 44, 54]. Having treatment in Malaysia is also supported by inexpensive transport costs compared to travel to Jakarta by plane which is 3–5 times more expensive. Reliable transport was also a contributing factor for patients seeking medical treatment overseas due to the convenience it afforded them [35, 38, 44]. Similarly, the reliability of services from health agencies overseas in connecting patients with foreign healthcare providers played a significant role in supporting Indonesian patients’ medical tourism and were reported to have accelerated medical tourism growth in countries, such as Malaysia, Singapore and Thailand [73, 74]. Health agencies were acknowledged to have added values to services, such as arranging pre- and post-treatment, travel arrangements, and scheduling tours in destination countries which increased the appeal of international medical tourism [73, 74]. Our findings indicate that Indonesian patients participate in international medical tourism due to having limited access to adequate quality healthcare within the borders of Indonesia. Improvements in the access, coverage, and quality of healthcare throughout Indonesia, (specifically in less urban areas) may reduce the occurrence of international medical tourism and improve patient perception of local health services.

High cost of medical treatment in hospitals in Indonesia was another common theme discussed in the majority of studies [20, 36–38, 44, 45, 51–53, 60, 49]. Medical treatment in Jakarta, for example, was considered more expensive than in Malaysia which is well known as the most preferred international medical travel destination due to its excellent service and cost affordability [15]. Our findings suggest that healthcare facilities with modern technologies are also available in some hospitals in big cities in Indonesia, such as Jakarta, East Java, West Java, and Central Java, however some studies reported issues in the quality of medical services and treatment being offered [44, 45]. This seemed to have resulted in an increased tendency for Indonesian patients to travel internationally to seek medical treatment and a second opinion from doctors. Moreover, different diagnostic results and faster recovery time received overseas undoubtedly have increased suspicion and distrust in medical treatment and doctors within Indonesia. Studies reported this was due to inaccurate diagnoses, ineffective medicines, incomprehensive assessment, and patients receiving inconsistent explanations regarding diseases [75, 76]. Such negative experiences have shown to have implications on both interpersonal trust in doctors who provide treatment to patients and institutional trust, particularly with the education system that trained the doctors [77, 78]. This in turn created negative perceptions towards the country’s health system.

Findings of this review have suggested similarities in religion (Islam) and culture (Malay and Chinese) were also factors that strengthened Indonesian patients’ preferences for medical treatment in other countries, such as Malaysia and Singapore [36, 47]. In addition, the growing level of dissatisfaction towards healthcare services in public or government owned hospitals has also become the underlying reason for many Indonesian people from upper class or secure economic backgrounds seeking medical treatment overseas. Our findings strengthen previous reports suggesting a lower satisfaction of patients towards healthcare service and treatment in public hospitals compared to private hospitals in Indonesia [79, 80]. The findings imply the need for the improvement of healthcare systems, medical treatments, and service delivery within the Indonesian public hospital sector.

### Implication for future intervention

This study emphasises the importance of prioritising the improvement of domestic health systems within Indonesia, particularly within periphery areas. This includes ensuring the equitable distribution of quality healthcare facilities, medical equipment, technology, and the fostering of a strong national healthcare workforce. Increasing the number of medical specialists within Indonesia and improving standards of care nationwide (and not just in urban areas) may promote engagement with Indonesian medical services over international ones. It is anticipated that such improvements would result in increased local service utilisation and reduced medical tourism as patients regain trust in the healthcare system within Indonesia. These findings could also be used to inform Indonesian healthcare workers on patients’ perceptions and concern with care.

### Implication for future study

This review suggests that there have been very limited studies involving Indonesian health workers or doctors in peripheral areas. Also, there have been very limited studies involving patients from Jakarta and other regions in Java that have contributed more than 50% of Indonesian patients taking medical tourism. None of the included studies involved policy makers from the Indonesian government and private sectors to explore their perspectives on the increased medical tourism among Indonesians. As there have been millions of Indonesian people travelling overseas for medical treatments, there is a need for further studies exploring the continuity and management of care for the patients returning home to Indonesia. Future studies that address all these aspects are recommended as the results can be used to inform and improve health policy and system and healthcare practice and delivery in Indonesia.

### Strength and limitation of the study

Although there are many studies on international medical travel among Indonesian patients, to our knowledge, this is the first scoping review on international medical travel taken by Indonesians. The use of several databases for data search helped researchers identify a broad range of themes on this topic involving Indonesian patients. However, as this review only included articles published in English and Indonesia, we may have missed studies on this topic reported in other languages.

## Conclusion

The review presents a range of supporting factors for Indonesian patients taking international medical travel, including the availability of health services, medical specialities, and person-centred care in other countries; region adjacency, transport, and health agency; affordability of medical treatment; religious and socio-cultural factors; and reasons patients reported distrust in Indonesian doctors. The findings indicate improvements in the Indonesian health system are necessary if the increasing rates of international medical tourism taken by Indonesian people is to change. Addressing the factors identified in this scoping review through avenues including policy may increase people’s satisfaction and trust towards health care and treatment in Indonesia, thereby reduce the number of Indonesian people taking medical tourism. The findings also indicate the need for establishment of international standard hospitals.

### Electronic supplementary material

Below is the link to the electronic supplementary material.

Supplementary Material 1

Supplementary Material 2

Supplementary Material 3

## Data Availability

All data generated or analysed during this study are included in this published article and its supplementary information files.
