# Supplementary material for: Medical tourism among Indonesians: a scoping review
Source: BMC Health Serv Res. 2024 Jan 10;24:49. doi: 10.1186/s12913-023-10528-1 (PMC10782758; doi:10.1186/s12913-023-10528-1)
Supplement: Supplementary file 1 — Supplementary Material 1 [file 12913_2023_10528_MOESM1_ESM.docx]

**Supplementary File 1: Description of the search terms used for each database.**

**Medline**

| ID | SEARCH | RESULT |
| --- | --- | --- |
| S1 | “International medical travel” | 15 |
| S2 | “medical tourism” | 1,580 |
| S3 | “health tourism” | 1,214 |
| S4 | “health travel” | 70 |
| S5 | (S1 OR S2 OR S3 OR S4) | 70 |
| S6 | Facilitators | 34,336 |
| S7 | "supporting factors" | 192 |
| S8 | reasons | 376,478 |
| S9 | "push factors" | 176 |
| S10 | "pull factors" | 263 |
| S11 | (S6 OR S7 OR S8 OR S9 OR S10) | 263 |
| S12 | patients | 8,586,146 |
| S13 | travellers | 6,144 |
| S14 | (S12 OR S13) | (S12 OR S13) |
| S15 | Indonesia* | 47,454 |
| S16 | Indonesian | 8,321 |
| S17 | (S15 OR S16) | 8,321 |
| S18 | (S15 OR S16)) AND (S5 AND S11 AND S14 AND S17)  ( “International medical travel” OR “medical tourism” OR “health tourism” OR “health travel” ) AND ( Indonesia OR Indonesian ) AND ( patients OR travellers ) | 7 |

**PubMed**

| ID | SEARCH | RESULT |
| --- | --- | --- |
| #1 | “International medical travel” | 15 |
| #2 | “medical tourism” | 1,559 |
| #3 | “health tourism” | 288 |
| #4 | “health travel” | 99 |
| #5 | (#1 OR #2 OR #3 OR #4) | 1,878 |
| #6 | Facilitators | [704,637](https://pubmed.ncbi.nlm.nih.gov/?term=Facilitators&sort=relevance) |
| #7 | "supporting factors" | [194](https://pubmed.ncbi.nlm.nih.gov/?term=%22supporting+factors%22+&sort=relevance) |
| #8 | reasons | [503,625](https://pubmed.ncbi.nlm.nih.gov/?term=reasons+&sort=relevance) |
| #9 | "push factors" | [176](https://pubmed.ncbi.nlm.nih.gov/?term=%22push+factors%22+&sort=relevance) |
| #10 | "pull factors" | [262](https://pubmed.ncbi.nlm.nih.gov/?term=%22pull+factors%22+&sort=relevance) |
| #11 | (#6 OR #7 OR #8 OR #9 OR #10) | [1,194,186](https://pubmed.ncbi.nlm.nih.gov/?term=%28%28%28%28Facilitators%29+OR+%28%22supporting+factors%22%29%29+OR+%28reasons%29%29+OR+%28%22push+factors%22%29%29+OR+%28%22pull+factors%22%29&sort=) |
| #12 | patients | [8,535,885](https://pubmed.ncbi.nlm.nih.gov/?term=patients&sort=relevance) |
| #13 | travellers | [130,420](https://pubmed.ncbi.nlm.nih.gov/?term=travellers&sort=relevance) |
| #14 | (#12 OR #13) | [8,620,097](https://pubmed.ncbi.nlm.nih.gov/?term=%28patients%29+OR+%28travellers%29&sort=) |
| #15 | Indonesia* | [46,390](https://pubmed.ncbi.nlm.nih.gov/?term=Indonesia%2A+&sort=relevance) |
| #16 | Indonesian | [8,393](https://pubmed.ncbi.nlm.nih.gov/?term=Indonesian&sort=relevance) |
| #17 | (#15 OR #16) | [46,390](https://pubmed.ncbi.nlm.nih.gov/?term=%28Indonesia%2A%29+OR+%28Indonesian%29&sort=) |
| #18 | (#15 OR #16)) AND (S# AND #11 AND #14 AND #17)  **(("International medical travel" OR "medical tourism" OR "health tourism" OR "health travel") AND (Indonesia OR Indonesian)) AND (Patients OR travellers)** | 9 |

**Scopus**

"International medical travel" OR "medical tourism" OR "health tourism" OR "health travel" AND facilitators OR "supporting factors" OR reasons OR "push factors" OR "pull factors" AND patients OR travellers AND indonesia* OR indonesian

Result 188

**ProQuest**

("International medical travel" OR "medical tourism" OR "health tourism" OR "health travel") AND (Facilitators OR "supporting factors" OR reasons OR "push factors" OR "pull factors") AND (Patients OR travellers) AND (Indonesia* OR Indonesian)

Result 337

**Wiley Online Library**

“International medical travel” OR “medical tourism” OR “health tourism” OR “health travel”" anywhere and "Facilitators OR "supporting factors" OR reasons OR "push factors" OR "pull factors"" anywhere and "Patients OR travellers" anywhere and "Indonesia* OR Indonesian" anywhere

Result 104
