# Supplementary material for: Medical tourism among Indonesians: a scoping review
Source: BMC Health Serv Res. 2024 Jan 10;24:49. doi: 10.1186/s12913-023-10528-1 (PMC10782758; doi:10.1186/s12913-023-10528-1)
Supplement: Supplementary file 2 — Supplementary Material 2 [file 12913_2023_10528_MOESM2_ESM.docx]

**Supplementary File 2: Characteristics of the included studies (Data extraction)**

| **Author/year** | **Study location** | **Aims** | **Study design** | **Analysis** | **Findings/ Main themes** |
| --- | --- | --- | --- | --- | --- |
| Abd Manaf et al, 2014 [1] | Malaysia | To explore the perception of international patients visiting Malaysia as a medical tourism destination country, as well as overall patient satisfaction, perceived value and future intention for repeat treatment and services. | Quantitative study | Regression analysis | Availability of health facilities and medical specialists:   - High standard level of medical staff - High standard level of medical facilities - International recognised hospitals - Recognised reputation of physicians - Preference of privacy and confidentiality   Region adjacency, transport and health agency   - Ease of accessibility - Ease of visa and immigration procedures - Ease of travel arrangements   Affordability of medical treatment   - Reasonable price and significant amount of money saved - Less expensive medical treatment - Opportunity for person who has no medical insurance   Socio-cultural factors   - No language barriers - Friendliness and helpfulness of the local people |
| Abdullah et al 2019[2] | Malaysia | To unpack factors attracting Indonesian medical tourists to Penang and employed a qualitative methodology in collecting information focusing only on Indonesian medical tourists. | Qualitative study   - Face to face interview | Thematic analysis | Availability of health services, medical specialities   - More organised hospitals and provided faster services   Region adjacency and transport   - Medan, Aceh, and Kalimantan are closer to Malaysia than to Jakarta or Java Island - Reliable public transport - Direct flights available   Affordability of treatment   - Cost for consultation, surgery, operations, and medicine are cheaper than in Indonesia. - Cheaper price of goods in Malaysia than in Indonesia   Reasons patients reported distrust in Indonesia doctors   - Effectiveness and accuracy of medical prescription higher in Malaysia - Higher accuracy of medical treatment in Malaysia   Socio-cultural factors   - Doctors and nurses can speak Indonesian language - Having family and friends living overseas |
| Adiwidjaja 2012 [3] | Indonesia | To describe the factors that encourage patients to travel from Indonesia to Singapore, Singapore's health-care reform at home and to investigate hospitals that provide excellent service to both countries. | Quantitative | Cross-sectional study | Availability of health services and medical specialists:   - Quality of treatment - Internationally recognised hospitals - Qualified medical staffs   Region adjacency and transport   - Medan and Aceh are closer to Singapore and Malaysia than to Jakarta - Reliable transport   Affordability of medical treatment   - Surgery in Malaysia is cheaper than in Indonesia   Reasons patients reported distrust in Indonesia doctors   - Visiting Singaporean doctors for second opinion of diagnosis and having medical treatment |
| Angela et al 2020 [4] | Indonesia | To segment Indonesian medical tourists based on push and pull travel motivation constructs | Quantitative study | Cross-sectional survey | Availability of health services, medical specialities and person-centred care   - Sophisticated and more modern health facilities - Excellent medical services - More responsive to patients   Reasons patients reported distrust in Indonesia doctors   - More specialists and trusted doctors in Malaysia - Improved patients’ recovery if being treated by doctors in Malaysia   Affordability of medical treatment   - Treatment costs match with the quality of treatment   Region adjacency and transport   - Some border areas in Indonesia are closer to Malaysia than to Java Island - Affordable flights - Sufficient local transportation - Adequate accommodation   Socio-cultural factors   - Language similarity - Culture similarity |
| Bennet L, Pangestu M 2017 [5] | Indonesia | To explore the preferences and decision-making processes of 15 married infertile Indonesian couples of high socioeconomic status, regarding intra-regional reproductive travel in Southeast Asia. | Qualitative/Ethnographic narratives   - Face to face interview | Thematic analysis | Availability of health services, medical specialities, and person-centred care   - Limited access to assisted reproduction technologies (ART) in Indonesia - Strict regulation on ART, sperm, and egg donor in Indonesia - Excellent health system   Transport   - Ease to cross border to Malaysia, Singapore, and Thailand   Affordability of medical treatment   - Minimal difference in cost between Malaysia and Thailand but more expensive in Singapore   Religious and socio-cultural factors   - Chinese ethnic patients visited Singapore as they have relatives and friends there - Feeling comfort being treated by Chinese doctors - Feeling comfort being treated by Muslim doctors   Reasons patients reported distrust in Indonesia doctors   - Patients believed that would receive better treatment or surgery overseas than in Indonesia |
| Budiwan, V. 2016 [6] | Indonesia | To investigate the understanding of a good service among Indonesian patients who have had medical treatment in Singapore. | Qualitative study   - Face to face interview | Thematic analysis | Availability of health services and medical specialities   - Knowledge and skills of medical staffs overseas - Less drugs prescribed compared to Indonesia - Sophisticated medical facilities and hospital hygiene   Person-centred care   - Honest and transparent - Keep communicating with patients and building interpersonal relationship - Medical staff are more responsive and friendly   Reasons patients reported distrust in Indonesia doctors   - Precise diagnose in Singapore - Felt safer when having treatment overseas   Affordability of medical treatment   - The cost is comparable - Some treatments are cheaper than in Indonesia |
| Chee HL & Whittaker A 2020 [7] | Indonesia | To explore moral reasons to take international medical travel and attracting factors in Malaysia | Qualitative study   - Face to face interview | Thematic analysis | Availability of health services and medical specialities   - High quality medical facilities - High qualified medical staffs   Region adjacency, transport, and health agency   - Aceh, Sumatra, and Kalimantan are closer to Malaysia than to Jakarta   Reasons patients reported distrust in Indonesia doctors   - Do not want to take risk with Indonesian doctors - The belief that doctors overseas have more experience - Concern about Indonesian doctors’ incompetence, negligence and unethical practices   Affordability of medical treatment   - Medical treatment in Malaysia is cheaper than in Indonesia |
| Chee HL et al 2019 [8] | Malaysia | To investigate international medical travel between Indonesia and Malaysia through the conceptual lens of sociality, transnational social space and therapeutic mobilities. | Qualitative study   - Face to face interview | Thematic analysis | Availability of health services and medical specialities   - Offering medical check-up packaged - Qualified medical staffs   Region adjacency, health agency and transport   - Medan and Aceh are closer to Malaysia than to Jakarta - Health agencies promote Malaysian health services in Indonesia - Health agencies arranged accommodation, transport, money exchange, and money transfer - Ease of access to Malaysia - Affordable transport in Malaysia   Socio-cultural factors   - Health staffs in Malaysia could speak Chinese and Indonesian language - Culture similarity |
| Kumar J & Hussia K 2016 [9] | Malaysia | To assess the factors for medical tourism destination selection from medical tourist’s perspective and to determine the satisfaction level of medical tourists in Malaysia | Quantitative study | Cross-sectional survey | Availability of health services and medical specialities   - Modern health facilities - High qualified medical staffs   Region adjacency and transport   - Aceh and Medan are closer to Malaysia than to Jakarta or Java Island - Ease to get flight - Reliable transport   Affordability of medical treatment   - The cost of medical treatment in Malaysia is cheaper than in Indonesia   Socio-cultural factors   - Language similarity either Chinese and Indonesian language - Culture similarity |
| Kwary 2019 [10] | Indonesia | to examine the influence of motivation and hospital reputation regarding the intention to make a medical visit to  Malaysia. | Quantitative |  | Availability of health services and medical specialities   - High chance to recover overseas - Modern health facilities - Hygienic environment - Qualified and experienced medical staffs - Hospitals hold international reputation   Transport   - Flights easy to access - Reliable transport in Malaysia   Affordability of medical treatment   - Medical treatment in Malaysia is cheaper than in Indonesia - Affordable accommodation |
| Manaf et al 2015 [11] | Indonesia | To examine service quality, perceived value, overall satisfaction and future intention among medical tourists who seek treatment in Malaysian private hospitals. | Quantitative study | Cross-sectional survey | Availability of health services, medical specialities, and person-centred care   - Quality medical treatment with reasonable price - Medical treatment delivered seen as high value - Administrative service quality - Short waiting lists   Region adjacency   - Medan is closer to Malaysia than to Jakarta or Java Island   Affordability of medical treatment   - The cost of treatment in Malaysia is cheaper than in Singapore and Thailand |
| Mahendradhata 2019 [12] | Indonesia | To inform policymakers in weighing the potential benefits and risks of promoting healthcare tourism in resource-constrained settings, such as Indonesia, by reviewing the lessons learned from healthcare tourism development, particularly in the region. | Review | Review | Availability of health services and medical specialities   - Experience of rapid services and results - Lack of modern health facilities in Indonesia - Lack of qualified medical staffs in Indonesia   Reasons patients reported distrust in Indonesia doctors   - Accurate diagnosis by doctors in Malaysia   Affordable medical treatment   - Spending less money in Malaysia than in Indonesia |
| Musa G et al 2012 [13] | Malaysia | To examine the demographic profile, travel motivation, healthcare consumption, and expenditure behaviour among patients in Kuala Lumpur. | Quantitative study | Cross-sectional survey | Availability of health services, medical specialities, and person-centred care   - Internationally recognised for quality standard - Waiting list is short - Clean and hygienic environment - Modern health facilities - Personal touch by doctors   Region adjacency and transport   - Aceh, Medan, and Kalimantan are closer to Malaysia than to Jakarta or Java Island - Ease to get and extend visa - Reliable transport in Malaysia   Affordability of medical treatment   - The cost is cheaper in Malaysia than in Indonesia   Religious and socio-cultural factors   - Similar religion background - Language similarity such as English, Chinese and Melayu - Culture similarity - Having relatives and friends overseas |
| Morissan 2021 [14] | Indonesia | To find out which factors, between the word of mouth (WoM) or marketing promotion programs (MPP), that drive motivation (push and pull); to explore the influence of travel motivation toward satisfaction, and to investigate the satisfaction effect of medical tourists with loyalty. | Quantitative | Cross-sectional survey | Availability of health services, medical specialities and person-centred care   - Quality of medical treatment in Malaysia - Satisfaction with medical services in Malaysia   Affordability of medical treatment   - The cost of medical services in Malaysia is cheaper than in Indonesia   Religious and socio-cultural factors   - Language similarity - Culture similarity - Same religion |
| Nasution et al 2020 [15] | Malaysia | To analyze the comparison of direct and indirect costs of medical tourism patients to Penang Island, Malaysia. | Quantitative study | Cross-sectional survey | Availability of health services and medical specialities, and person-centred care   - Modern health facilities and technology in hospitals in Malaysia - Doctors in Malaysia more attentive in caring patients   Region adjacency and transport   - Ease of access to Malaysia - Medan, Aceh, and Kalimantan are closer to Malaysia than to Jakarta or Java Island   Reasons patients reported distrust in Indonesia doctors   - Receiving more accurate diagnosis by doctors in Malaysia - Medicines given to patients are more appropriate and effective   Affordability of medical treatment   - Lower cost in Malaysia than in Indonesia   Socio-cultural factors   - Language similarity - Culture similarity |
| Nuryani A 2020 [16] | Indonesia | To review the speakers’ presentation on the international webinar about medical tourism in Indonesia | Conference review | Review | Availability of health services, medical specialities, and person-centred care   - Lack of specialists or appropriate doctors   Reasons patients reported distrust in Indonesia doctors   - Accurate diagnosis - Effective medications   Affordability of treatment   - Looking for cheaper options with better quality |
| Ormond M 2014[17] | Indonesia | To explore impacts international medical travels on medical travellers' home contexts | Qualitative study   - Face to face interview | Descriptive study | Availability of health services, medical specialities, and person-centred care   - Lack of health facilities in border areas of Indonesia - Lack of qualified medical staffs in border areas of Indonesia - Poor quality pharmaceuticals   Region adjacency and transport   - Closer to Malaysia than to Jakarta or java island - Reliable transport in Malaysia   Reasons patients reported distrust in Indonesia doctors   - Inaccurate diagnosis by Indonesian doctors - Poor communication - Incomplete explanation - Ineffective medicines given   Affordability of treatment   - The cost is cheaper in Malaysia than in Indonesia |
| Ormond, M 2015 [18] | Malaysia | To explore how transport operators and infrastructure respond and adjust to the embodied specificities of the growing market’s access and travel needs. | Qualitative study   - Face to face interview | Thematic analysis | Health agency   - Health agencies arrange accommodation, transport, and hospital booking   Region adjacency and transport   - Medan, Aceh, and Kalimantan are closer to Malaysia than to Jakarta or Java Island - Reliable transport in Malaysia   Affordability of treatment   - The cost of treatment is cheaper in Malaysia than in Indonesia   Reasons patients reported distrust in Indonesia doctors   - Accurate diagnosis in Malaysia - Appropriate and effective medicines given in Malaysia |
| Ormond, M & Sulianti D 2017 [19] | Indonesia | To explore Indonesian medical travellers’ motivations, preparations and practices. | Qualitative study   - Face to face interview | Thematic analysis | Availability of health services, medical specialities   - Lack of qualified medical staffs in borders areas in Indonesia - Lack of modern health facilities in border areas of Indonesia - Little incentives for doctors   Person-centred care   - Doctors in Malaysia and Singapore apply person care: keep communicating with patients and short waiting to meet doctors   Region adjacency and transport   - Malaysia and Singapore are closer to some areas of Indonesia compared to Jakarta or Java Island   Reasons patients reported distrust in Indonesia doctors   - Doctors in Malaysia are more open, honest, friendly. - Doctors in Malaysia provide clear and comprehensive explanation - Inaccurate diagnosis by Indonesian doctors - Ineffective medicines given |
| Ratnasari RT et al 2022 [20] | Indonesia | To identify potential development of medical services with the concept of medical tourism in Indonesia by comparing with Malaysia. | Mixed method (quantitative and qualitative) | Thematic analysis | Availability of health services and medical specialities   - Modern health facilities in Malaysia - Quality of treatment in Malaysia   Region adjacency and health agency   - Some areas of Indonesia are closer to Malaysia than to Jakarta or Java island - Health agencies offered holistic services for medical specialists, appointment, accommodation, transport, and tourist attractions   Reasons patients reported distrust in Indonesia doctors   - Inaccurate diagnosis in Indonesia - Clear and comprehensive explanation in Malaysia   Affordability of medical treatment   - The cost of treatment is cheaper in Malaysia than in Indonesia |
| Saragih HS and Jonathan P 2019 [21] | Malaysia | To examine Indonesian consumers’ through the use of behavioural lenses to examine their medical tourism experiences in Malaysia, its neighbouring country. | Quantitative study | structural equation modelling (SEM) | Availability of health services and medical specialities   - Highly trained medical staff - Quality of medical treatment - Ease to find information about medical specialists   Region adjacency   - Some areas in Indonesia are closer to Malaysia than to Java Island   Affordability of treatment and transport   - Cost-effective medical treatment in Malaysia   Socio-cultural factors   - No language barriers |
| Whittaker et al 2017 [22] | Indonesia | To explore historical interconnections in travel for medical treatments. | Qualitative study   - Face to face interview | Thematic analysis | Region adjacency and transport   - Sumatra and Aceh are closer to Malaysia than to Jakarta - Availability of direct flight and ferry   Reasons patients reported distrust in Indonesia doctors   - Lack of trust to quality of care of Indonesian doctors - Inaccurate diagnosis by Indonesian doctors - Lack of experiences among Indonesian doctors - Indonesian doctors have substandard ethics - Prescription of unnecessary medicines by Indonesian doctors   Affordability of treatment and transport   - Cost effective in Malaysia - Private hospitals in Indonesia charged the same in Malaysia   Religious and socio-cultural factors   - Cultural similarity between Indonesia and Malaysia - More ethnic Chinese people in Malaysia - More Muslim doctors in Malaysia |
| Widiyastuti et al., 2023 [23] | Indonesia | To examine the factors that influence Entikong District residents' use of Sarawak health services. | Quantitative | Cross-sectional study | Availability of health services and medical specialities   - Lack of health facilities in Entikong - Lack of doctors in Entikong - Quality of medical treatment in Malaysia - Positive experiences from other people having treatment in Malaysia   Region adjacency and transport   - Entikong (Indonesia) is closer to Malaysia than to Jakarta or Java Island - Reliable transport to Malaysia than to other neighbour districts in Kalimantan |
| Yeoh et al 2013 [24] | Malaysia | To determine the demographics of the medical tourists that are visiting Malaysia for various medical treatments. | Quantitative study | Cross-sectional survey | Availability of health services, medical specialities   - Quality of medical treatment - Positive experiences from other having treatment in Malaysia - Referral from Indonesian doctors |
| Zain MD et al 2022 [25] | Indonesia | To explore the Indonesian tourists’ demand for medical tourism services in Malaysia. | Qualitative study   - Face to face interview | Thematic analysis | Availability of health services medical specialities, and person-centred care   - Administrative inconvenience - Unnecessary medicines - Long waiting time - Inadequate medical specialists - Positive experiences from others heaving medical treatment in Malaysia   Reasons patients reported distrust in Indonesia doctors   - Inaccurate diagnosis by Indonesian doctors - Ineffective medicines given - Slow health improvements if being treated by Indonesian doctors - Openness, communication and transparency of hospital practices in Malaysia |
